# Unveiling the Enigma: Multiple Primary Neoplasms Mimicking Metastatic Disease

**DOI:** 10.7759/cureus.72145

**Published:** 2024-10-22

**Authors:** Ali Zafar Sheikh

**Affiliations:** 1 Radiology, Shaukat Khanum Memorial Cancer Hospital and Research Centre, Lahore, PAK

**Keywords:** astrocytoma, intra-axial supratentorial brain lesion, metachronous malignancies, metastatic deposit, papillary thyroid carcinoma, synchronous malignancies

## Abstract

Papillary thyroid cancer (PTC) is the most common type of thyroid malignancy, typically associated with a favorable prognosis due to its slow progression and high treatability. Standard treatment often involves surgical resection followed by radioactive iodine therapy, which significantly reduces the risk of recurrence. However, despite these effective interventions, rare and unexpected complications can arise, posing significant diagnostic and therapeutic challenges.

While PTC rarely metastasizes beyond regional lymph nodes, distant metastases, particularly to the brain, are exceedingly uncommon and often signal a more aggressive disease course. The presentation of brain metastasis from PTC is rare. Concurrently, primary brain tumors such as astrocytoma can present diagnostic dilemmas, particularly when they arise in patients with a history of other malignancies.

This case report aims to shed light on the complexities and challenges faced in diagnosing and managing a patient with a history of PTC who later presents with neurological symptoms suggestive of intracranial pathology. By reviewing the existing literature and discussing this unique presentation, we aim to contribute to the understanding of rare metastatic patterns and the critical importance of comprehensive diagnostic evaluations in patients with previous malignancies.

## Introduction

Multiple primary cancers, though rare, have been widely studied and documented in the medical literature, with reported incidence rates varying between 0.3% and 4.3% [1]. This phenomenon, known as multiple primary neoplasms (MPNs), involves the occurrence of two or more distinct malignant tumors either simultaneously or sequentially. When these tumors appear at the same time, they are classified as synchronous, whereas if they develop at different points in time, they are termed metachronous [2,3]. The classification of MPNs is important because it helps to understand the underlying mechanisms behind their development. They are typically divided into three primary categories: treatment-associated, where cancer treatment itself leads to subsequent tumors; syndrome-related, where genetic predispositions are responsible for multiple tumors; and tumors that arise from common etiological factors, such as environmental exposures or lifestyle-related risks [2,3].

While brain metastasis is a recognized complication in papillary thyroid carcinoma (PTC), it remains relatively uncommon, occurring in only a small percentage of cases [4-7]. Even more unusual is the co-occurrence of synchronous or metachronous primary brain tumors in patients diagnosed with PTC [1,8]. These cases are considered exceptional, making them rare occurrences in both clinical reports and the broader medical literature. Understanding the interplay between PTC and brain tumors, whether through common genetic pathways or unique metastatic behavior, presents a fascinating and complex challenge in oncology.

## Case presentation

**Figure 1:**
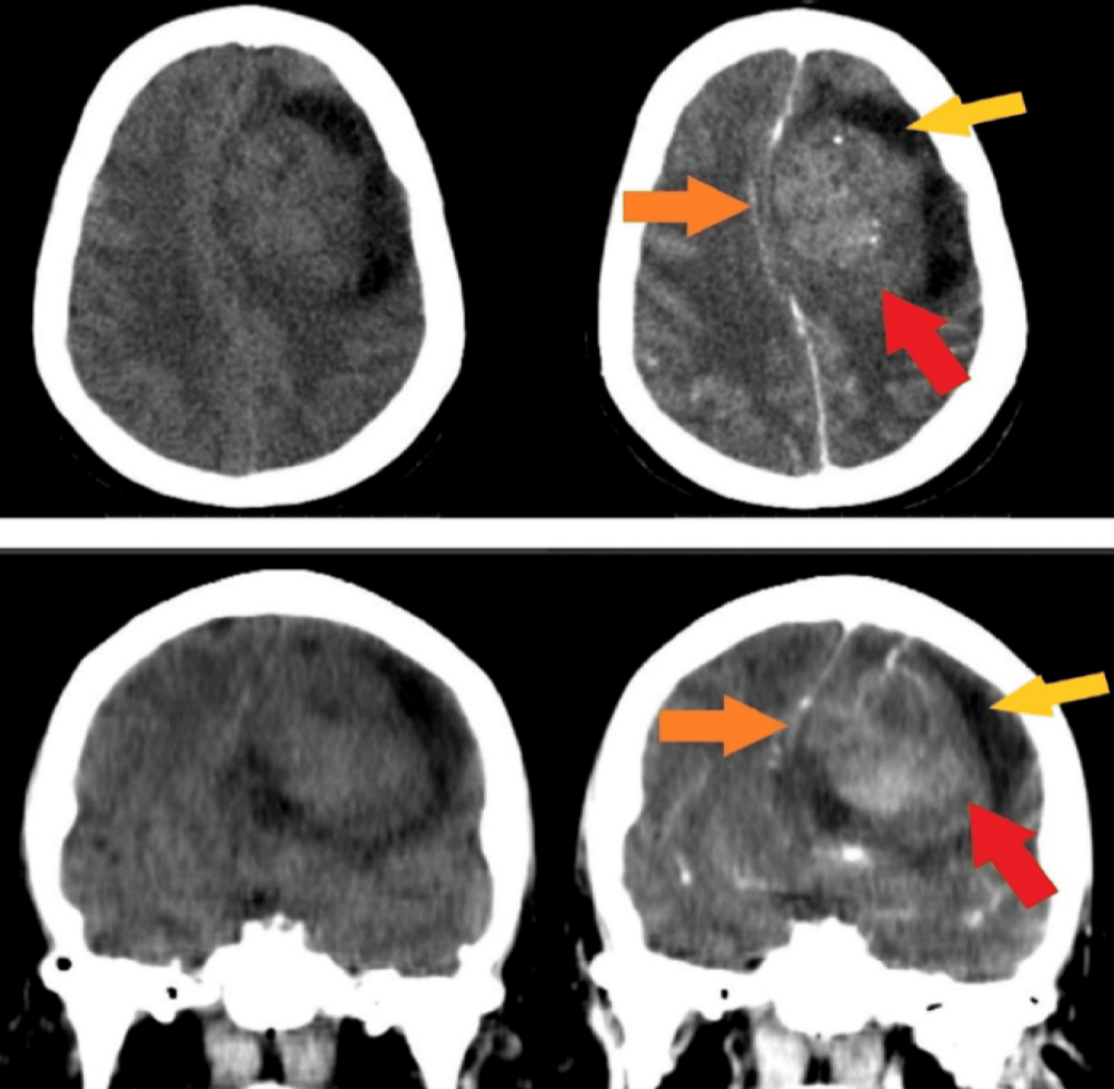
CT brain without and with contrast enhancement; axial (upper row) and coronal sections (lower row) Solitary enhancing, space-occupying lesion (red arrows) in the left frontal lobe with midline shift (orange arrows) and surrounding edema (yellow arrows)

**Figure 2:**
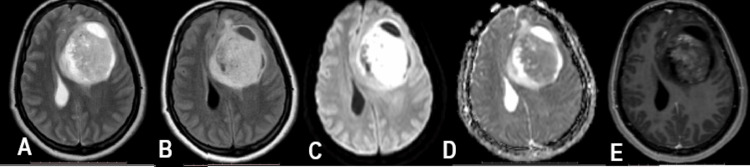
MRI brain, axial slices; T2 Weighted Image (T2WI), Fluid Attenuation Inversion Recovery (FLAIR), Diffusion Weighted Image (DWI), Apparent Diffusion Coefficient (ADC) and Contrast Enhanced T1 Images (C+T1WI), sequences. Predominantly solid intra-axial, supratentorial lesion in the left frontal lobe causing mass effect in the form of midline shift and compression upon the ipsilateral lateral ventricle. It is returning hyperintense T2 and FLAIR signals (A & B), demonstrating diffusion restriction on DWI and ADC (C & D) and post-contrast enhancement (E)

A 37-year-old female patient was diagnosed with a papillary thyroid cancer epicenter in the left lobe with vascular invasion in December 2020. She underwent a left-sided lobectomy in January 2021. The residual tissue was also excised in April 2021, and later she underwent radioactive iodine therapy. In September 2022, she presented with a few months' history of amnesia and a headache that increased in intensity and later became associated with vomiting. She underwent CT with and without contrast, demonstrating a space-occupying lesion in the left frontal lobe with midline shift, while rest of the study was unremarkable for any other lesion or meningeal disease (Figure 1). It was followed by contrast-enhanced MRI brain, demonstrating solitary, solid intra-axial, supratentorial abnormal signal intensity lesion in the left frontal lobe, the abnormal signals were T2 and fluid-attenuated inversion recovery (FLAIR) hyperintense, the lesion demonstrated diffusion restriction and post-contrast enhancement (Figure 2). The CT chest, abdomen and pelvis was unremarkable for metastatic disease.

**Figure 3:**
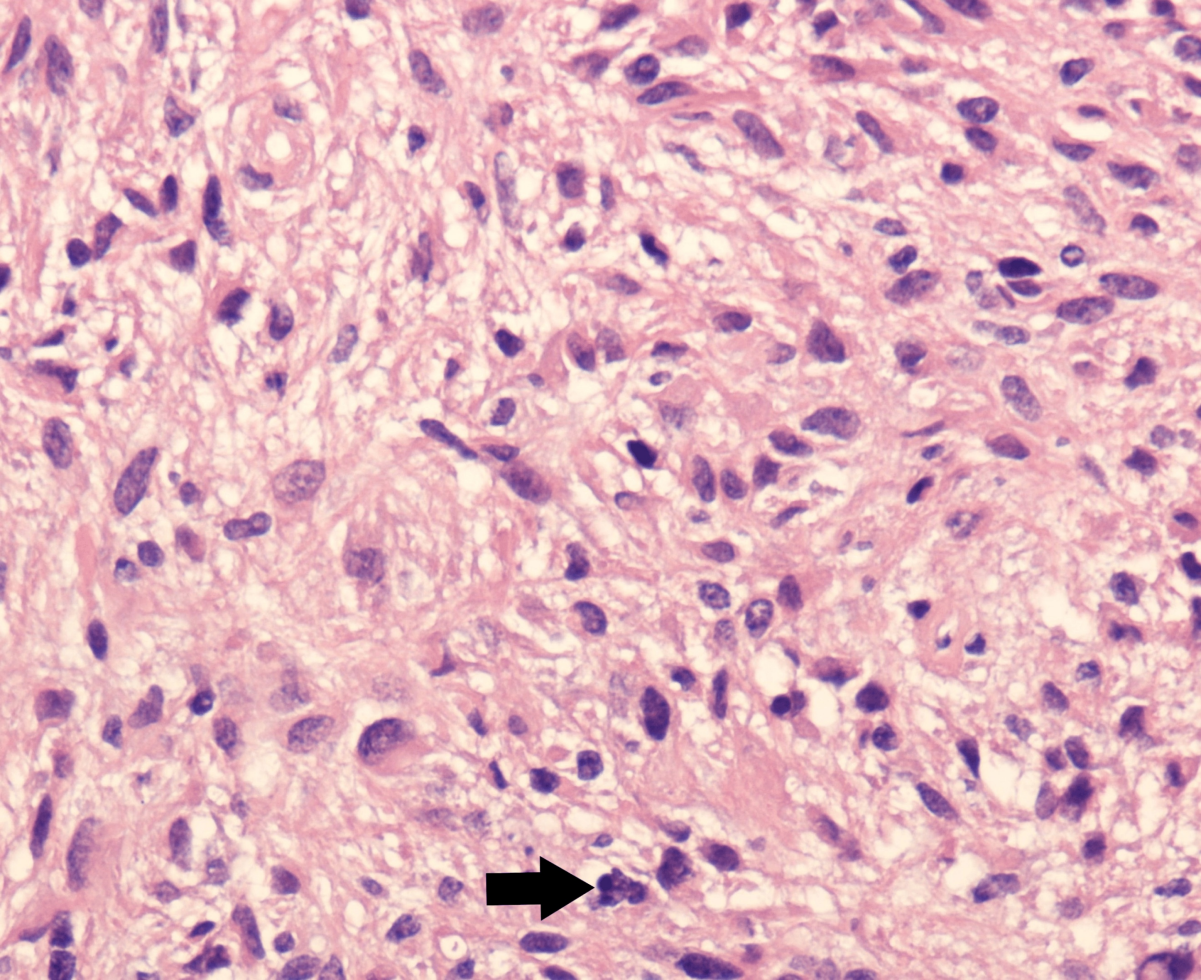
Image of excisional biopsy-tissue sample under microscope Abnormal appearing astrocytes of variable shapes and sizes (pleomorphism), with variable appearance of nuclei (nuclear atypia) and single mitotic figure (black arrow)

The case was discussed in the multidisciplinary team conference, and considering it to be a solitary metastatic deposit, radiotherapy was suggested to decrease the tumor bulk. The patient was reassessed in November 2022 after completion of radiotherapy, and the tumor turned out to be stable in the interim. She underwent surgical excision on December 27, 2022, and the excised-out lesion was subjected to biopsy, which turned out to be astrocytoma grade 2, IDH mutant (Figure 3).

## Discussion

The reported incidence of multiple primary cancers ranges from 0.3% to 4.3%, firmly establishing its status as a well-researched phenomenon [1]. The term multiple primary neoplasms is used to refer to two or more tumors that exist at the same time or over some time, provided that the following conditions are met: each tumor must be malignant, exhibit distinct characteristics, and not be a metastatic deposit of the other tumor [2]. Based on the timing of their identification, these two tumors can be classified as either synchronous or metachronous tumors. Synchronous tumors are characterized by the presence of two or more independent tumors that are identified either simultaneously or within six months. On the other hand, metachronous tumors are defined by a time gap greater than six months between the identification of each tumor [2,3]. MPNs can be broadly classified into three primary groups based on their main causal factors. The first group comprises neoplasms that are associated with treatments or therapies. The second group involves cases related to specific syndromes. Lastly, the third group encompasses neoplasms that may share common etiological factors, such as genetic predisposition or exposure to similar environmental factors [2,3]. Furthermore, it's worth noting that the occurrence of two or more cancers can also happen purely by chance, independent of any specific causal factors [2]. While not exceedingly prevalent, brain metastasis in PTC is not remarkably uncommon [4-7]. However, the occurrence of synchronous or metachronous brain tumors with PTC is exceptionally rare [1,8].

## Conclusions

This case of a 37-year-old female with both papillary thyroid carcinoma and an astrocytoma highlights the importance of thorough diagnostic evaluations when multiple primary neoplasms are suspected. The initial assumption of brain metastasis was revised to a primary brain tumor after histopathological examination, emphasizing the need for comprehensive assessment and a multidisciplinary approach in managing complex cancer cases. This case underscores the critical need for awareness and diligence in distinguishing between primary and metastatic lesions to ensure accurate diagnosis and effective treatment.

## References

[1] Makarov A, Loskutova K, Innokentyeva A, Makarova E, Korosteleva L (2012). Synchronous papillary thyroid cancer and astrocytoma: case report. Endocr Abstr.

[2] Sakellakis M, Peroukides S, Iconomou G, Boumpoucheropoulos S, Kalofonos H (2014). Multiple primary malignancies: a report of two cases. Chin J Cancer Res.

[3] Rayan A, Ashraf AA, Bakri HA (2018). Multiple primary malignancies: metastatic renal with early breast and endometrial cancers: a case report. J Cancer Ther.

[4] Miranda ER, Padrão EL, Silva BC, De Marco L, Sarquis MS (2010). Papillary thyroid carcinoma with brain metastases: an unusual 10-year-survival case. Thyroid.

[5] Knight J, Kingham M, Price SA, Bodi I, Lavrador JP (2022). Delayed brain metastasis after 17-years from papillary thyroid cancer without local recurrence: case report and literature review. J Surg Case Rep.

[6] Kim SS, Kim SM, Park M, Suh SH, Ahn SJ (2021). Clinico-radiological features of brain metastases from thyroid cancer. Medicine (Baltimore).

[7] Emamhadi MR, Alijani B, Chabok SY, Behzadnia H, Dehghani S (2015). Brain metastasis from papillary thyroid carcinoma in a seventy-three-year-old man. Arch Neurosci.

[8] Palaniandy K, Kamarudin Z, Wong YP, Mohamed Mukari SA, Jiau WX, Bakar AA (2022). Case report: triple whammy: synchronous radiotherapy induced glioblastoma multiforme and papillary thyroid cancer following nasopharyngeal carcinoma. Front Oncol.

